# Diuretics are Similar to Losartan on Echocardiographic Target-Organ Damage in Stage I Hypertension. PREVER-Treatment Study

**DOI:** 10.5935/abc.20180249

**Published:** 2019-01

**Authors:** Carolina Bertoluci, Murilo Foppa, Angela Barreto Santiago Santos, Sandra C. Fuchs, Flávio Danni Fuchs

**Affiliations:** 1 Serviço de Cardiologia - Hospital de Clínicas de Porto Alegre, Porto Alegre, RS - Brazil; 2 Programa de Pós-graduação em Cardiologia da Faculdade de Medicina da Universidade Federal do Rio Grande do Sul, Porto Alegre, RS - Brazil

**Keywords:** Angiotensin-Converting Enzyme Inhibitors, Anihypertensive Agents, Antihypertensive Agents/therapy, Blood Pressure, Hypertension/complications, Hypertension/therapy, Calcium Channel, Echocardiography

## Abstract

Blood pressure (BP)-lowering therapy improves left ventricular (LV) parameters of hypertensive target-organ damage in stage II hypertension, but whether there is a drug-class difference in echocardiographic parameters in stage I hypertension patients is less often studied. In the PREVER treatment study, where individuals with stage I hypertension were randomized for treatment with diuretics (chlorthalidone/amiloride) or losartan, 110 participants accepted to participate in a sub-study, where two-dimensional echocardiograms were performed at baseline and after 18 months of antihypertensive treatment. As in the general study, systolic BP reduction was similar with diuretics or with losartan. Echocardiographic parameters showed small but significant changes in both treatment groups, with a favorable LV remodeling with antihypertensive treatment for 18 months when target blood pressure was achieved either with chlorthalidone/amiloride or with losartan as the initial treatment strategy. In conclusion, even in stage I hypertension, blood pressure reduction is associated with improvement in echocardiographic parameters, either with diuretics or losartan as first-drug regimens.

## Introduction

Heart failure with preserved ejection fraction (HFPEF) is an increasingly prevalent condition where hypertension has an important role.^1^ Echocardiography identifies increased left ventricular mass (LVM),^2^ LV concentric remodeling,^3^ left atrial (LA) enlargement and diastolic dysfunction,^4^ which are used to diagnose HFPEF,^1^ and are independently associated with cardiovascular events.

Blood pressure (BP)-lowering treatment improves diastolic function and reduces LVM, LA size, especially in stage II hypertension, but the degree of benefit may be different among medications.^5^ Whether there are differences in echocardiographic parameters with different antihypertensive drug classes in stage I hypertension is less often studied.

The present study was undertaken to compare the effects of chlorthalidone/amiloride versus losartan on echocardiographic evidence of hypertensive consequences in patients with stage I hypertension.

## Methods

This is an echocardiographic sub-study at a single center of the PREVER-treatment study,^6^ a multicenter double-blind randomized controlled trial (RCT) comparing chlorthalidone together with amiloride versus losartan for the management of stage I hypertension as the first option in the management of stage I hypertension.

Population, methods and results of the PREVER-treatment study are described in detail elsewhere.^6^ In summary, all eligible participants of the PREVER-treatment study were aged 30 to 70 years old, with stage I hypertension according to the Eighth Joint National Committee (JNC 8) guidelines on hypertension (systolic BP between 140 and 159 or diastolic BP 90 and 99 mmHg)^7^ and were not taking antihypertensive medication. They were submitted to a pre-enrollment lifestyle intervention phase; if BP remained inadequately controlled after 3 months of lifestyle intervention, they were enrolled in the RCT. Participants were randomly assigned in a 1:1 ratio to a chlorthalidone/amiloride 12.5/2.5 mg combination pill or to losartan 50 mg. A reassessment was performed every 3 months and, if necessary, treatment was scaled up with open label add-on BP drugs according to the protocol. The final visit occurred after 18 months of follow-up.

Transthoracic echocardiography was obtained at baseline and after 18 months of treatment. All echocardiographic examinations were performed using the same equipment (Envisor C HD or HD 11, Philips) with a standard multifrequency sectorial transducer by 2 trained cardiologists blinded to trial information and treatment allocation, following a previously described standardized protocol.^8^ Echocardiographic studies were blindly read by a single physician using a dedicated workstation (Image Arena version 4 - TomTec, Germany). Measurements were performed in accordance with international society guidelines.^9^ The study was approved by the institution's human research committee and informed consent was obtained from each patient.

Comparisons between the initial and final echocardiographic measurements in each treatment group were assessed by paired t-tests. Comparisons between the differences in treatment groups were assessed by independent-sample t-tests. An overall linear model was used to adjust echocardiographic outcomes for mean blood pressure variation, baseline echocardiographic parameter and time between randomization and echocardiographic examination. Intraobserver reproducibility was evaluated in 20 randomly chosen studies using intraclass correlation coefficient, and varied between 0.99 and 0.67, with the lowest reproducibility found for the posterior wall thickness measurement.

## Results

Of the 655 participants of the PREVER-treatment study, 230 participants from Hospital de Clínicas de Porto Alegre center were invited to participate in the echocardiographic evaluation, of which 133 participants were willing to participate, and 110 underwent the echocardiograms at baseline and after 18 months of follow-up.

Baseline demographic and clinical characteristics are shown in Table 1. Systolic blood pressure (SPB) was lower in the losartan group than in the main study, but it was similar between patients receiving diuretics and losartan who underwent echocardiograms. All other baseline characteristics were similar between the treatment groups and the main study group, including previous use of antihypertensive drug (diuretics: 71.4%, losartan: 65%, p = 0.47).

**Table 1:** Baseline clinical and demographic characteristics of participants by treatment group

	PREVER-treatment study		Echo substudy	
	Diuretics (n = 333)	Losartan (n = 322)	Diuretics (n = 56)	Losartan (n = 54)
Sex (male)	167 (50.2)	167 (51.9)	34 (60.7)	28 (51.9)
Age (years)	53.9 ± 8.4	54.7 ± 7.9	55.5 ± 7.6	54.1 ± 8.3
BMI (kg/m²)	29.1 ± 5.0	28.8 ± 4.7	28.5 ± 4.4	28.5 ± 4.3
SBP (mmHg)	142.6 ± 7.1	142.1 ± 6.5	142.2 ± 8.2	139.4 ± 6.0
DBP (mmHg)	89.7 ± 6.3	89.4 ± 6.1	90.6 ± 5.9	90.2 ± 5.6

Diuretics: chlorthalidone/amiloride; BMI: body mass index; SBP: systolic blood pressure; DBP: diastolic blood pressure. Data are expressed as mean ± SD or number (%).

As shown in Table 2, there was no significant difference between the treatment groups regarding the final SBP. There was a similar proportion of patients receiving full dose of amlodipine (10 mg per day) after 18 months of follow-up in both treatment groups (5,3% in diuretics group, 9,2% in losartan group, p = 0.43).

**Table 2:** Adjusted differences in blood pressure and echocardiographic parameters between diuretics (chlorthalidone/amiloride) and losartan treatment groups*

Variable	Drug	Baseline	18-Month Follow-Up	Change from baseline	p	Between group change	p	Adjusted between group change**	p
SBP (mmHg)	Diuretics	142.2 ± 8.2	129.8 ± 10.0	-12.4 ± 11.1	< 0.001	2.7	0.18	1.18	0.51
	Losartan	139.4 ± 6.0	129.7 ± 8.7	-9.7 ± 9.4	< 0.001				
DPB (mmHg)	Diuretics	90.6 ± 5.9	83.7 ± 7.0	-6.8 ± 5.9	< 0.001	1.2	0.33	0.77	0.49
	Losartan	90.2 ± 5.6	82.1 ± 6.8	-8.0 ± 6.6	< 0.001				
LVMI (g/m^2^)	Diuretics	84 ± 17	81 ± 19	-3 ± 16	0.11	1.78	0.48	3.84	0.14
	Losartan	82 ± 17	77 ± 16	-4 ± 14	0.02				
IVST (mm)	Diuretics	10.0 ± 1.2	9.7 ± 1.3	-0.3 ± 1.2	0.03	0.34	0.13	0.60	0.009
	Losartan	10.0 ± 1.1	9.4 ± 1.2	-0.7 ± 1.1	< 0.001				
PWT (mm)	Diuretics	10.1 ± 1.1	9.5 ± 1.1	-0.6 ± 3.3	< 0.001	-0.13	0.47	0.16	0.38
	Losartan	9.8 ± 1.1	9.4 ± 1.0	-0.46 ± 1.1	0.002				
RWT	Diuretics	0.45 ± 0.06	0.42 ± 0.05	-0.04 ± 0.06	< 0.001	-0.009	0.47	0.007	0.53
	Losartan	0.44 ± 0.06	0.41 ± 0.05	-0.03 ± 0.07	0.006				
LAVI (ml/m^2^)	Diuretics	25.4 ± 6.5	24.1 ± 6.9	-1.4 ± 6.2	0.12	1.24	0.28	0.26	0.83
	Losartan	28.2 ± 7.8	25.7 ± 5.9	-2.6 ± 5.2	0.001				
Medial E/e’ ratio	Diuretics	8.1 ± 2.1	8.5 ± 2.6	0.42 ± 2.52	0.23	0.61	0.21	0.22	0.65
	Losartan	8.8 ± 2.3	8.6 ± 2.3	-0.19 ± 2.39	0.57				
EDT (ms)	Diuretics	229.2 ± 47.4	252.2 ± 67.2	23.0 ± 63.0	0.01	11.0	0.37	13.33	0.34
	Losartan	230.0 ± 45.4	243.8 ± 66.9	12.0 ± 64.2	0.19				

* Diuretics: n = 56; Losartan: n = 54.

** Analysis of covariance adjusted for mean blood pressure variation, corresponding baseline echocardiographic parameter and time between randomization and echocardiographic exam. Diuretics: chlorthalidone/amiloride; SBP: systolic blood pressure; DBP: diastolic blood pressure; LVMI: left ventricular mass index; IVST: interventricular septum thickness; PWT: posterior wall thickness; RWT: relative wall thickness; LAVI: left atrial volume index; EDT: E-wave deceleration time. Data are expressed as mean ± SD.

Baseline echocardiographic parameters were similar among the groups (Table 2), except for LA volume index (LAVI) which was higher in the losartan group (28.2 ± 7.8 mL/m² vs 25.4 ± 6.5 mL/m², p < 0.05). After 18 months of treatment, there was a significant reduction in interventricular septal thickness (IVST), posterior wall thickness (PWT) and relative wall thickness (RWT), with a significant rise in E-wave deceleration time (EDT) in the diuretics group; in the losartan group, there was a significant reduction in LA volume index (LAVI), LVM index (LVMI), IVST, PWT and RWT (Table 2).

After adjustment for mean blood pressure variation, baseline echocardiographic parameter and time between randomization and echocardiographic examination, individuals in the losartan group had a greater interventricular septal thickness reduction (-0.7 ± 1.1 mm *vs*. -0.3 ± 1.2 mm; adjusted difference: 0.6 mm; p=0.009). However, this reduction was not sufficient to translate into differences in geometric patterns or diastolic function parameters between the treatment groups.

## Discussion

This study shows that, in stage I hypertension, LV mass and LA size reductions, and changes in diastolic function parameters were similar with chlorthalidone/amiloride or with losartan treatment for 18 months.

Detection of target-organ damage is important for an adequate estimate of prognosis of the hypertensive patient. Increased LV mass and hypertrophy independently predict cardiovascular events. Despite concerns about echocardiographic variability,^10^ it is the first-line imaging study for LV mass evaluation. In our study, to increase reproducibility of measurements, all studies were blindly read to visit and treatment allocation, and the paired analysis of data allowed the measurement of the intrinsic variation for each participant.

Two large studies directly compared different antihypertensive drug classes. The TOMHS study, in the pre-angiotensin receptor antagonist (ARB) era, evaluated 844 patients with stage I hypertension randomized for non-pharmacological treatment and chlorthalidone, acebutolol, amlodipine, enalapril, doxazosin or placebo.^11^ Only chlorthalidone promoted regression of LVH compared to placebo in 12 months (-4.8g vs -18.2g; p = 0.04), with no difference observed in 48 months. It is important to note that, during follow-up, 33% of patients on the placebo group were prescribed active medication.

The LIFE substudy evaluated 960 patients with a higher SBP (160-200 mmHg) randomized for losartan or atenolol.^12^ After 5 years, LVM showed greater reduction with losartan than with atenolol (-21.7 g vs -17.7 g; p = 0.01), although BP reduction was similar. In this study, LVM reduction was also more pronounced during the first 12 months of treatment. It should be noted that more patients on the losartan group were also using hydrochlorothiazide.

As far as we know, only one study directly compared diuretic (hydrochlorothiazide) and ARB (telmisartan) use in 69 patients with DBP of 90-114 mmHg, showing a higher reduction of LVM estimated by three-dimensional echocardiography with telmisartan (16 g *versus* 4 g in 12 months).^13^ It is noteworthy that ARB was used at a maximum dose and the diuretic at a low dose.

The results of our study are in line with the findings of a meta-analysis^5^ summarizing randomized comparative studies of antihypertensive treatment on LV mass regression in patients with stage II hypertension. There was less LV mass regression with beta-blockers, while diuretics, calcium channel blockers, angiotensin-converting enzyme inhibitors and ARB had similar effectiveness. We showed that there was no difference on LV mass regression after 18 months between a diuretic-based *versus* an ARB-based treatment of patients with stage I hypertension.

The study limitations should be acknowledged. The anticipated breach in randomization is not likely to have impacted the results, as demographic characteristics of the studied sample and the magnitude of SBP reduction were similar to those achieved in the whole sample study. Also, study power could be underestimated to find statistically significant differences in echocardiographic parameters between randomized treatments, as the PREVER-treatment study sample size was estimated for its primary endpoint. These potential limitations, however, reinforce the reported findings, which are even more noticeable if we consider the relatively low burden of hypertension organ damage, and the follow-up of only 18 months.

## Conclusion

In stage I hypertension, blood pressure reduction is associated with improvement in echocardiographic parameters of target-organ damage, with a favorable LV remodeling achieved with either diuretics or losartan as the initial treatment strategy.
